# Membrane lymphotoxin-α_2_β is a novel tumor necrosis factor (TNF) receptor 2 (TNFR2) agonist

**DOI:** 10.1038/s41419-021-03633-8

**Published:** 2021-04-06

**Authors:** Kirstin Kucka, Isabell Lang, Tengyu Zhang, Daniela Siegmund, Juliane Medler, Harald Wajant

**Affiliations:** Division of Molecular Internal Medicine, Department of Internal Medicine II, University Hospital Würzburg, Würzburg, Germany

**Keywords:** Cytokines, Signal transduction

## Abstract

In the early 1990s, it has been described that LTα and LTβ form LTα_2_β and LTαβ_2_ heterotrimers, which bind to TNFR1 and LTβR, respectively. Afterwards, the LTαβ_2_–LTβR system has been intensively studied while the LTα_2_β–TNFR1 interaction has been ignored to date, presumably due to the fact that at the time of identification of the LTα_2_β–TNFR1 interaction one knew already two ligands for TNFR1, namely TNF and LTα. Here, we show that LTα_2_β interacts not only with TNFR1 but also with TNFR2. We furthermore demonstrate that membrane-bound LTα_2_β (memLTα_2_β), despite its asymmetric structure, stimulates TNFR1 and TNFR2 signaling. Not surprising in view of its ability to interact with TNFR2, LTα_2_β is inhibited by Etanercept, which is approved for the treatment of rheumatoid arthritis and also inhibits TNF and LTα.

## Introduction

**Fig. 1:**
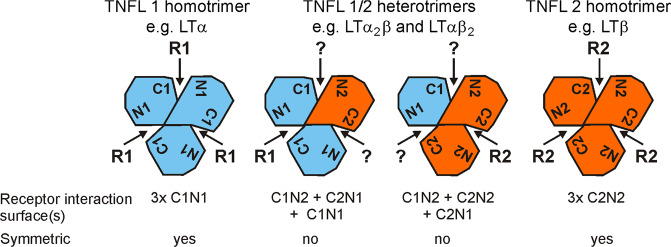
Receptor interaction surfaces of homo- and heterotrimeric TNFL molecules. Homo- and heterotrimeric ligand molecules assembled from protomers of two different types of TNFSF ligands (TNFL1 and TNFL2) can form four different (C1N1, C1N2, C2N1, C2N2) interaction areas for the TNFRs (R1, R2) recognized by TNFL1 and TNFL2. While the two homotrimeric variants possess each three identical (3xC1N1 or 3xC2N2), symmetrically organized receptor interaction surfaces, heterotrimeric TNFLs have inevitably three different receptor interaction areas (C1N1 + C1N2 + C2N1 or C1N2 + C2N1 + C2N2). See text for details.

The ligands of the tumor necrosis factor (TNF) superfamily (TNFSF) are characterized by a C-terminal TNF homology domain (THD), which promotes (i) the assembly into homotrimeric, and in a few cases also heterotrimeric, molecules and (ii) the binding to receptors of the TNF receptor superfamily (TNFRSF)^1,2^. The TNFSF ligands (TNFLs) form a structurally comparatively homogeneous protein family^1,2^. With the exception of lymphotoxin-α (LTα), all TNFLs are initially expressed as type II transmembrane proteins in which the extracellular THD is linked to the transmembrane domain and the intracellular domain by a “stalk” region. Some TNFLs also occur as soluble variants that arise from the membrane-bound molecules by processing in the “stalk” region. Since the soluble TNFLs, including LTα, contain the THD, these molecules also occur as trimers. Although LTα protomers do not have a transmembrane domain, they can be membrane-bound by the formation of heterotrimeric molecules with lymphotoxin-β (LTβ)^3–6^. Indeed, in addition to the very-well-investigated homotrimeric molecule variants, which with the exception of LTβ have been described for all ligands of the TNFSF, a few cases are known in which two different ligands of the TNFSF assemble to form stable heterotrimeric molecules. In addition to the LTα–LTβ heterotrimers already mentioned, Baff–APRIL and Baff–TWEAK heterotrimers have also been described^7,8^. Due to their intrinsic asymmetry, the heterotrimeric ligands of the TNFSF inevitably have three different receptor interaction surfaces (Fig. 1). One of these three interaction areas corresponds to one of the homotypic interaction areas that can be found in the two corresponding homotrimeric TNFLs (Fig. 1). The two other heterotypic receptor interaction surfaces (i.e., formed by different protomers) are different and common to the two possible heterotrimeric ligand configurations (Fig. 1).

In the initial publications on the production of the LTα–LTβ heterotrimers, it was described that LTα_2_β and LTαβ_2_ heterotrimers are formed after ectopic coexpression of LTα and soluble LTβ^4,6^. Systematic FACS analysis with a panel of different LTα antibodies also argued for the existence of two different forms of membrane-bound and thus LTβ-bound LTα^3^. The early studies with recombinantly produced LTα–LTβ heterotrimers reported only a binding of LTα_2_β to TNFR1 but found no binding to the LTβR, which interacts with LTαβ_2_ but not with LTα homotrimers or TNF. The LTα_2_β species of LTα–LTβ heterotrimers has therefore not received any further attention to date. It has not been investigated whether LTα_2_β activates TNFR1 signaling and it is also unclear whether LTα_2_β binds to and activates TNFR2.

Here, we show that LTα_2_β is able to trigger TNFR1 signaling despite having reduced valency compared to LTα and TNF homotrimers to this receptor. We also demonstrate that soluble and transmembrane LTα_2_β interact with TNFR2. More intriguingly, we found that transmembrane LTα_2_β robustly activates TNFR2 signaling. We have thus identified transmembrane LTα_2_β as a novel TNFR2 agonist.

## Results

### TNFR1 and TNFR2 interact with LTα_2_β heterotrimers

**Fig. 2:**
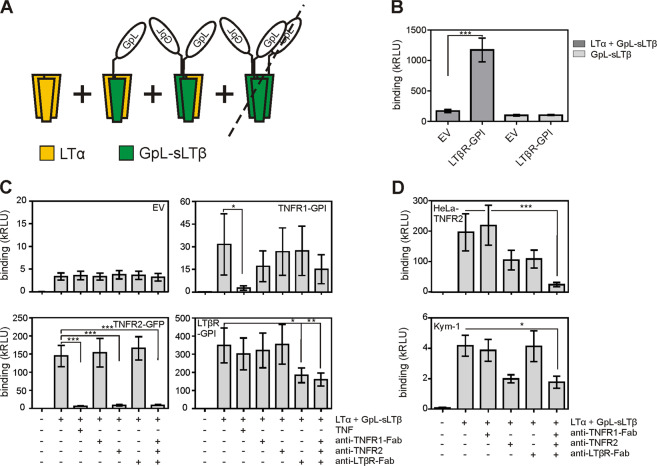
Heterotrimeric LTαβ species interact with LTβR, TNFR1, and TNFR2. **A** Scheme of possible trimers formed upon coexpression of LTα and a GpL fusion protein of soluble LTβ (GpL-sLTβ). Please note, LTβ trimers are expressed but do not bind to LTβR. **B** HEK293T cells were transiently transfected with empty vector (EV) or an expression plasmid encoding LTβRed-GPI. Cells were then incubated for 1 h with 1 µg/ml of cell culture supernatant, transfected with GpL-sLTβ or a mixture of LTα and GpL-sLTβ-encoding expressions plasmids. Cell bound molecules were quantified by measuring GpL activity. Shown are results from three independent experiments. **C** HEK293T cells were transiently transfected with empty vector (EV) or expression plasmids encoding TNFR1-GPI, TNFR2-GFP, or LTβRed-GPI and were preincubated the next day as indicated for 30 min with 10 µg/ml TNF, 10 µg/ml of a TNFR2-specific antibody, TNFR1- or LTβR-specific Fabs. Cells were then incubated for 1 h with a cell culture supernatant of cells transfected with a 1:1 mixture of LTα and GpL-sLTβ-encoding expressions plasmid (final ligand concentration 20 ng/ml). Finally, cell bound molecules were quantified by measuring GpL activity. Shown are results from 10 (EV), 3 (TNFR1-GPI), 6 (TNFR2-GPI) and 8 (LTβR-GPI) independent experiments. **D** HeLa-TNFR2 and Kym-1 cells were preincubated for 30 min with 10 µg/ml anti-TNFR1-Fab, anti-TNFR2, anti-LTβR-Fab, or a combination of all three molecules and were then evaluated with respect to binding of GpL-sLTβ-containing ligand species as in (C). Shown are the results from six different experiments. ***p < 0.001; **p < 0.01; *p < 0.05; repeated-measures ANOVA.

To initially confirm the reported binding of LTα_2_β heterotrimers to TNFR1, we coexpressed soluble LTα along with soluble LTβ (sLTβ) bearing an N-terminal *Gaussia princeps* luciferase (GpL) reporter domain and analyzed the binding of the resulting cell culture supernatants with respect to TNFR1 and LTβR binding (Fig. 2A–C). We included in this analysis also TNFR2 for which an interaction with LTα_2_β heterotrimers has not been evaluated so far. To determine the binding of GpL-sLTβ-containing ligand species to TNFR1, TNFR2, and LTβR, HEK293T cells, which have no or only very low endogenous expression of these receptors, were transiently transfected with expression plasmids encoding a TNFR2–GFP fusion protein, glycophosphatidylinositol (GPI)-anchor-tagged variants of the ectodomains of TNFR1 (TNFR1ed-GPI) and LTβR (LTβRed-GPI) or empty vector (EV). In contrast to the EV-transfected cells, the HEK293T cells transfected with plasmids encoding the various TNFR variants all showed strong binding of GpL-sLTβ-containing ligand species (Fig. 2C). Binding of GpL-sLTβ-containing ligand species to TNFR1ed-GPI-expressing cells was efficiently blocked by soluble TNF and also reduced by a ligand-blocking anti-TNFR1-Fab, while a ligand-blocking anti-TNFR2 antibody and a ligand-blocking LTβR-specific Fab showed no effect (Fig. 2C). Similarly, binding of GpL-sLTβ-containing ligand species to TNFR2-GFP-expressing cells was almost completely prevented by soluble TNF and the ligand-blocking anti-TNFR2 antibody, while the blocking TNFR1- and LTβR-specific Fabs showed no inhibitory effect (Fig. 2C). Last but not least, neither TNF or TNFR1-specific Fab nor the blocking TNFR2-specific antibody interfered with binding of GpL-sLTβ-containing ligand species to the LTβRed-GPI-expressing transfectants while the ligand-blocking LTβR-specific Fab significantly reduced the binding (Fig. 2C). Since it is well established that LTαβ_2_ does not interact with other TNFRs and that LTβ homotrimers are not stable/functional (see also Fig. 2B), these data confirmed the formation of sLTα_2_β heterotrimers in LTα- and LTβ-coexpressing cells that can interact with TNFR1. Moreover, these data similarly argue that sLTα_2_β heterotrimers interact with TNFR2. To further substantiate the evidence of the interaction of TNFR2 with a GpL-sLTβ-containing ligand species, we analyzed binding to cells with stable TNFR2 expression. For this, we used stable HeLa-TNFR2 transfectants, which express app. 2000 TNFR1 molecules and 50,000 TNFR2 molecules^9^, and Kym-1 cells expressing app. 1000-3000 TNFR1 molecules and 30,000 TNFR2 molecules^10^. Again, there was strong binding of GpL-sLTβ-containing ligand species to these cells. In accordance with the quite different expression levels of TNFR1 and TNFR2, the anti-TNFR2 antibody but not the anti-TNFR1-Fab diminished binding of the GpL-sLTβ-containing ligand species (Fig. 2D). Blockade of endogenous LTβR in HeLa-TNFR2 cells with anti-LTβR-Fab also reduced binding and a mixture of all three blocking reagents significantly inhibited binding of the GpL-sLTβ-containing ligand species (Fig. 2D).

**Fig. 3:**
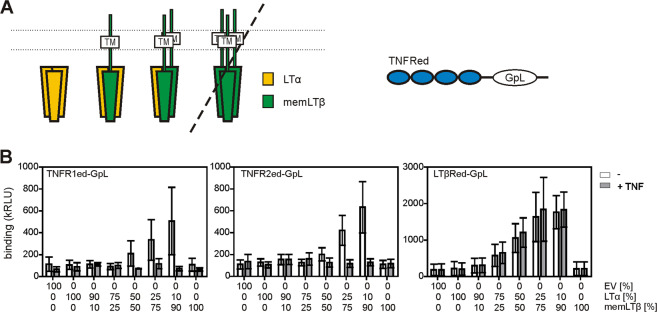
The soluble ectodomains of TNFR1, TNFR2, and LTβR bind to different cell-bound heteromeric LTαβ species. **A** Scheme of the TNFRed–GpL fusion proteins used in (B). **B** HEK293T cells were transfected with empty vector (EV) or a mixture of LTα- and memLTβ-encoding expression plasmids. Next day, binding of 500 ng/ml TNFR1ed–GpL, TNFR2ed–GpL, and LTβRed–GpL was analyzed in the presence and absence of 20 µg/ml TNF. Data shown are technical replicates of one representative experiment of three independent experiments.

Next, we wondered whether LTα and membrane LTβ form TNFR1- and TNFR2-interacting heteromers as well. To clarify this issue, we transiently coexpressed LTα and memLTβ in HEK293T cells and analyzed the binding of soluble TNFR fusion proteins consisting of the extracellular domain of TNFR1, TNFR2, and LTβR and a C-terminal GpL reporter domain (TNFR1ed–GpL, TNFR2ed–GpL, LTβRed–GpL; Fig. 3A). It turned out that all three TNFRed–GpL fusion proteins specifically bound to cells coexpressing LTα and memLTβ (Fig. 3B). The addition of an excess of soluble TNF inhibited the binding of the soluble TNFR1ed–GpL and TNFR2ed–GpL molecules to the LTα/memLTβ-cotransfected cells, but showed no effect on LTβRed–GpL binding (Fig. 3B). This is not only consistent with the established formation of LTβR-interacting membrane-bound LTαβ_2_ heterotrimers (memLTαβ_2_), but also indicates the interaction of membrane-bound LTα_2_β molecules (memLTα_2_β) with TNFR1 and TNFR2.

### Single-chain-encoded memLTα_2_β heterotrimers activate TNFR1 and TNFR2

**Fig. 4:**
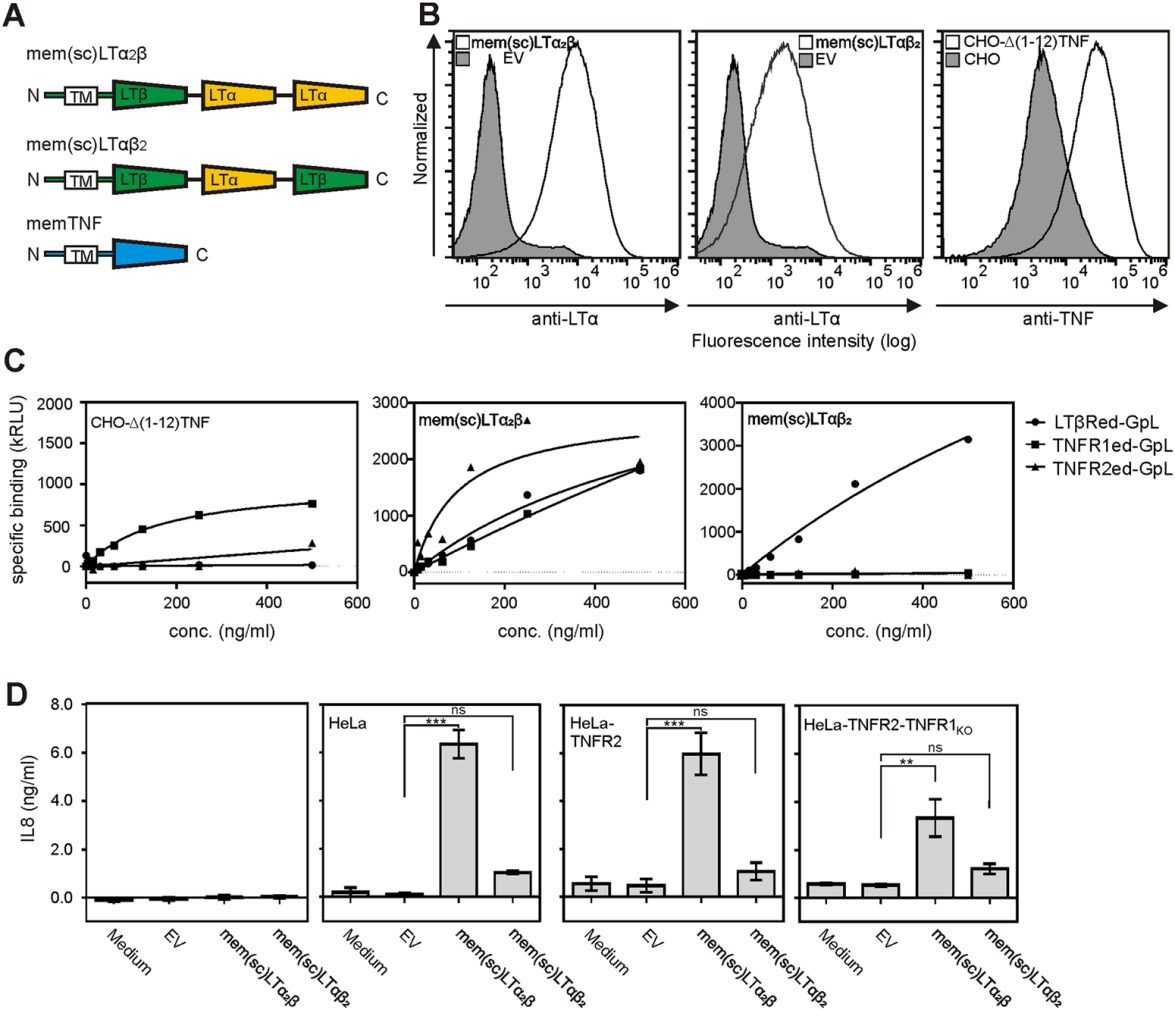
mem(sc)LTα_2_β but not mem(sc)LTαβ_2_ activate TNFR1 and TNFR2. **A** Scheme of single-chain encoded memLTαβ variants and memTNF. **B** Flow cytometry analysis of Flp-In HEK293 transfectants stably expressing mem(sc)LTα_2_β and mem(sc)LTαβ_2_ and of a CHO transfectant stably expressing a non-cleavable mutant of memTNF. Empty vector (EV) transfected cells served as negative controls. **C** The indicated Flp-In HEK293 and CHO-Δ(1-12)TNF cells were analyzed with respect to binding of TNFR1ed-GpL, TNFR2ed-GpL, and LTβRed-GpL. One representative experiment of three independent experiments are shown. **D** HeLa, HeLa-TNFR2, and HeLa-TNFR2-TNFR1_KO_ cells were cocultured 1:1 (50,000 cells each) with the indicated ligand transfectants. Cell culture supernatants were analyzed the next day for their IL8 content by ELISA. Shown are results from three different experiments. ***p < 0.001; **p < 0.01; n.s., not significant, repeated-measures ANOVA.

**Fig. 5:**
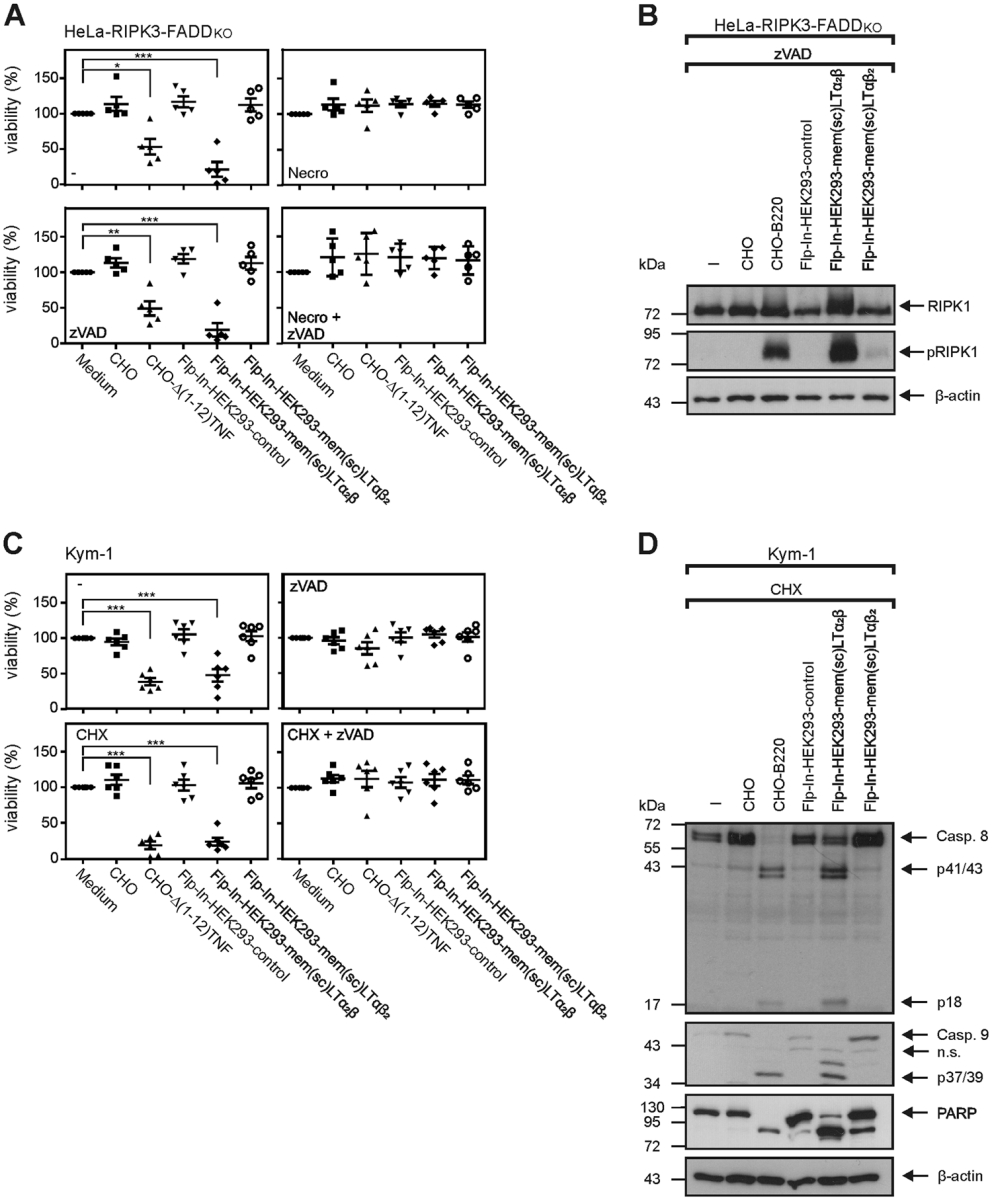
mem(sc)LTα_2_β induces necroptosis and apoptosis. **A**, **B** HeLa-RIPK3-FADD_KO_ cells were challenged with the indicated cell lines/transfectants 1:1 overnight (5 × 10^4^ cells each) (A) or for 8 h (1 × 10^6^ cells each) (B). Cell cultures were then analyzed by crystal violet staining for cellular viability (A) and by western blotting (B) for S166 phosphorylation of RIPK1. Where indicated the RIPK1 inhibitor nec-1 was added to prevent necroptosis, and the pan-caspase inhibitor zVAD was added to prove involvement of caspases. Shown in (A) are the results of five independent experiments. **B** shows one of two experiments with comparable results. **C**, **D** Kym-1 cells were challenged 1:1 with the indicated cell lines overnight (5 × 10^4^ cells each) and were then analyzed by crystal violet staining for cellular viability (C) and by western blotting (1 × 10^6^ cells each) (D) for processing of the indicated proteins. Where indicated the RIPK1 inhibitor nec-1 was added to prevent necroptosis and the protein synthesis inhibitor cycloheximide (CHX) was added for apoptosis sensitization. Shown in (C) are the results of six independent experiments. **D** shows one of two experiments with comparable results. ***p < 0.001; **p < 0.01; *p < 0.05; repeated-measures ANOVA.

To investigate in the following more specifically the interaction of memLTα_2_β and the two TNF receptors, we fused by genetic engineering a memLTβ protomer and two LTα protomers to obtain membrane-bound single-chain-encoded LTα_2_β (mem(sc)LTα_2_β). For comparison, we included, where appropriate, in our investigations single-chain-encoded memLTαβ_2_ (mem(sc)LTαβ_2_) and memTNF (Fig. 4A). Flp-In HEK293 cells, which express no endogenous LTβR or TNFR2 and only low levels of TNFR1 (Supplemental Fig. 1), were stably transfected with mem(sc)LTα_2_β and mem(sc)LTαβ_2_ encoding expression plasmids, resulting in clones with roughly comparable ligand expression (Fig. 4B, Supplemental Fig. 2). Flow cytometry showed a shift in the MFI by a factor of approximately 10 and 50 (Fig. 4B), which is in the range observed in reference 3 for endogenous expression of total LTα–LTβ heterotrimers. Even if the staining efficacy of antibodies varies to some extent, this suggests that the expression levels in the stable transfectants are not excessively high. We considered membrane TNF (memTNF)-expressing transfectants as a positive control for TNFR1 and TNFR2 binding. We used for this purpose CHO-Δ(1-12)TNF, a previously described^11^ stable CHO transfectant expressing a non-cleavable deletion mutant of membrane TNF (Fig. 4B). Cellular binding studies with soluble LTβRed–GpL, TNFR1ed–GpL, and TNFR2ed–GpL revealed exclusive interaction of mem(sc)LTαβ_2_ with LTβRed–GpL (Fig. 4C). In contrast, the mem(sc)LTα_2_β- and the memTNF-expressing cells bound TNFR1ed–GpL and TNFR2ed–GpL (Fig. 4C). Noteworthy, the mem(sc)LTα_2_β expressing transfectants showed also some binding of LTβRed–GpL (Fig. 4C). Next, we analyzed the ability of the mem(sc)LTα_2_β and mem(sc)LTαβ_2_ heterotrimers to trigger TNFR1 and/or TNFR2 activation. For this purpose, we cocultivated the Flp-In HEK293 mem(sc)LTα_2_β and Flp-In HEK293 mem(sc)LTαβ_2_ transfectants and the corresponding control cells with (i) HeLa cells, only expressing TNFR1, (ii) HeLa–TNFR2 transfectants^9^, expressing TNFR1 and TNFR2, and (iii) HeLa–TNFR2-TNFR1_KO_ cells, a derivative of HeLa–TNFR2 cells expressing only TNFR2 (Supplemental Fig. 3A, B). Exclusive stimulation of both, TNFR1 and TNFR2, result in IL8 production in HeLa cells what could be straightforwardly quantified by ELISA (Supplemental Fig. 3C). It turned out that mem(sc)LTαβ_2_-expressing cells triggered in all three HeLa variants a very minor IL8 response (Fig. 4D). This is in good accordance with the hardly detectable expression of the LTβR in HeLa cells. In contrast, mem(sc)LTα_2_β-expressing transfectants induced robust IL8 production in all three HeLa variants, indicating that this variant can stimulate TNFR1 as well as TNFR2 signaling (Fig. 4D). Upregulation of IL8 expression reflects activation of the classical NFκB pathway, which can be activated by TNFR1 and TNFR2. We also tested the ability of mem(sc)LTα_2_β to trigger necroptosis and apoptosis. We recently showed that FADD-deficient HeLa–RIPK3 transfectants are highly susceptible to TNFR1-induced necroptosis^12^. Cocultivation of these cells with the mem(sc)LTα_2_β-expressing transfectants resulted in cell death induction, which could be prevented by treatment with the RIPK1 inhibitor nec-1, which prevents necroptotic activation of RIPK1 (Fig. 5A). In contrast, as expected, the pan-caspase inhibitor zVAD showed no protective effect (Fig. 5A). We furthermore detected RIPK1 S166 phosphorylation, a hallmark of necroptotic signaling, in cocultures of HeLa-RIPK3 with mem(sc)LTα_2_β expressing cells but not in cocultures with Flp-In Hek293 control cells (Fig. 5B). Similarly, the mem(sc)LTα_2_β-expressing cells induced cell death and caspase activation in Kym-1 cells (Fig. 5C,D), a well-established cell system in which both TNF receptors cooperate to induce apoptosis^13,14^. Thus, despite its asymmetric nature and its reduced valency for TNFR1/2 binding, mem(sc)LTα_2_β is able to trigger TNFR1 and TNFR2 signaling. Not surprising that in view of these results, mem(sc)LTα_2_β-induced TNFR1 and TNFR2 activation was inhibited by Etanercept (Supplemental Fig. 4), a dimeric Fc fusion protein of the human TNFR2 ectodomain that is widely used in the clinic to treat rheumatoid arthritis.

## Discussion

The intrinsic asymmetry of heterotrimeric TNFLs results in three different receptor interaction surfaces (Fig. 1). TNFR1 and TNFR2 do not or only weakly bind LTαβ_2_. The latter do not share an identical receptor interaction area with LTα_3_ homotrimers but share with the LTα_2_β heterotrimer the two differing heterotypic receptor interaction surfaces formed between LTα und LTβ protomers in LTαβ heterotrimers (Fig. 1). Thus, it can be concluded that LTα_2_β bind TNFR2 only/preferentially with its single homotypic receptor interaction surface formed between the two LTα protomers. We also observed residual binding of GpL–s(sc)LTα_2_β to the LTβR. Again, this makes sense in the view of what is known about the interaction between LTαβ_2_ and the LTβR. Structural data of the complex between a single chain-encoded LTαβ_2_ heterotrimer and the LTβR showed that two molecules of the latter interact with the scLTαβ_2_ molecule: while one binds to the single homotypic receptor interaction surface formed between the two LTβ protomers, the second one binds to one of the two differing heterotypic receptor interaction surfaces^15^. Thus, it can be expected that LTα_2_β binds with one of its heterotypic receptor interaction surfaces to the LTβR which is in accordance with our binding data.

TNFR1 and TNFR2 are of paramount importance in the regulation of acquired and adaptive immunity and play accordingly a crucial role in the development and progression of autoimmune diseases, such as rheumatoid arthritis, multiple sclerosis, Crohn’s disease and psoriasis. Against this background, it would be interesting and important to know what relative contribution TNF, LTα, and LTα_2_β make in vivo to the biology and pathobiology of the two TNF receptors. However, this aspect is difficult to investigate with the tools and experimental approaches currently available. So, there is e.g., no antibody that allows to distinguish LTα_2_β from LTαβ_2_ and LTα_3_. In vivo studies to identify LTα_2_β-mediated effects furthermore are challenging because the knockout (but also the ectopic expression) of LTα or LTβ inevitably also affects LTαβ_2_ biology. Only the indirect study of memLTα_2_β-mediated TNFR2 activation is conceivable using knockout models. Under consideration of the fact that LTα does not activate TNFR2, the comparison of TNF knockout mice and TNF-TNFR2 double-deficient mice may provide insights into memLTα_2_β-induced TNFR2 activities, e.g., in context of the biology of regulatory T-cells where TNFR2 seems to play an important function^16^. For such studies, however, it would first have to be clarified whether murine LTα_2_β has receptor binding properties analogous to those of the human LTα_2_β examined in our study.

## Methods

### Cell lines and reagents

HEK293T, HeLa, HeLa-TNFR2, HeLa-TNFR2-TNFR1_KO_, HeLa-RIP3-FADD_KO_, Kym-1, and CHO cells, and a stable CHO transfectant expressing a non-cleavable mutant form of membrane TNF (CHO-Δ(1-12)TNF)^11^ cells were cultivated in RPMI 1640 medium (Sigma-Aldrich, Germany) with 10% fetal calf serum (FCS) (GIBCO). HEK-Flp In cells and all transfectants derived thereof were cultivated in DMEM medium (Sigma-Aldrich, Germany) with 10% FCS. For the HEK Flp In cells and not the transfectants, the medium was supplemented with 100 µg/ml Zeocin^TM^ (Thermo Fisher Scientific, MA, USA). Antibodies used in this study were purchased from the following suppliers: BD Biosciences, NJ, USA (anti-PARP, 551025; anti-RIPK1, 610459), Cell Signaling, MA, USA (anti-p-RIPK1, 65746 S; anti-Caspase-9, 9502 S), Enzo Life Sciences, Germany (anti-Caspase-8, ADI-AAM-118-E), Santa Cruz Biotechnology, Santa Cruz, CA, USA (anti-TNFβ (TNFβ = LTα) (E-6), sc-28345), Sigma-Aldrich, Germany (anti-mouse IgG (whole molecule), R-PE-labeled, P9670; anti-β-Aktin, A1978) and Thermo Fisher Scientific, MA, USA (anti-TNFα, PE-labeled, #12-7349-82). Expression plasmids encoding antibodies, antibody fragments, TNFRSF receptors, and the various TNFSF ligand variants were obtained by cloning corresponding DNA fragments and PCR amplicons or combinations thereof in the pCR3 expression vector (Invitrogen, Germany) or into pcDNA5/FRT (Thermo Fisher Scientific, MA, USA). The amino acid sequences of the resulting proteins are indicated in Supplemental Table I.

### Generating stable cell lines

Stable HEK293 transfectants expressing mem(sc)LTα_2_β and mem(sc)LTαβ_2_ were established using the Flp/FRT system. For this, Flp-In-HEK293 cells were cultivated in 10 cm tissue cell culture plates in DMEM supplemented with 10% FCS until 60–70% confluence had been reached. Then, for each plate, 1 ml of serum free DMEM medium was supplemented with 3 µg of the recombinase encoding plasmid pOG44 and 3 µg of pOG44 (negative control) or the empty vector plasmid pcDNA5/FRT or mem(sc)LTα_2_β and mem(sc)LTαβ_2_ encoding derivatives of pcDNA5/FRT. After dropwise addition of 18 µl of a 1 mg/ml polyethylenimine (PEI, Polyscience Inc., Warrington, USA) solution, the mixture was vortexed and incubated for 10 to 15 min at room temperature. Then, the medium on the Flp-In-HEK393 plates was replaced by the DNA/PEI-mixture and 7 ml of serum-free DMEM medium. Stable transfectants were selected by supplementing the medium next day with 75 µg/ml Hygromycin (Merck, Germany, 400051) and 10% FCS. When all cells were dead in the negative control, the surviving cells of the pcDNA/FRT, pcDNA/FRT-memLTα_2_β, and pcDNA/FRT-memLTαβ_2_ transfections were used for limited dilution. Finally, clones were analyzed by flow cytometry for expression of membrane-bound LTαβ heterotrimers.

### Expression, production, and purification of antibodies, antibody fragments, TNFRSF receptors, and TNFSF ligands

HEK293T cells were transiently transfected with the expression plasmid(s) of interest using polyethylenimine (PEI, Polyscience Inc., Warrington, USA) as described elsewhere^17,18,19^. Cells transfected with membrane-bound TNFRSF receptors or membrane-bound TNFSF ligands were used for binding studies and flow cytometry 1 or 2 days post transfection. For production of secreted proteins, all of which were tagged with a Flag epitope, 1 day post transfection the PEI/DNA-containing transfection medium was replaced by RPMI 1640 medium supplemented with 2% FCS. After 5–7 days supernatants were cleared from cellular debris by centrifugation (10 min, 4630 × *g*). Protein concentrations were determined by western blotting and comparison with a dilution series of a Flag-tagged protein standard of known concentration. Constructs containing a *Gaussia princeps* luciferase reporter domain for binding studies were used without further purification. The other proteins were purified by anti-Flag affinity chromatography as described elsewhere^17^. The purity and concentration of the purified proteins were controlled by SDS-PAGE, silver staining of the gel (12.5%) with the Pierce Silver Stain Kit (Thermo Fisher Scientific, MA, USA, 24612), and protein standard proteins of known concentrations and molecular weight (Amersham LMW Calibration Kit for SDS Electrophoresis, GE Healthcare).

### Binding studies

#### Binding of GpL-tagged soluble ligand variants to cell-expressed TNFRs

HEK293T cells transiently transfected with TNFR expression plasmids, HeLa-TNFR2, or Kym-1 cells were harvested and aliquoted in 1.5 ml safety-lock tubes (0.5–1.5 × 10^6^ cells/tube). Cells were then, when indicated, pretreated with blocking TNFR1-, TNFR2- or LTβR-specific antibodies or antibody fragments for 30 min at 37°C prior. Cells were incubated for 1 h with supernatants of HEK293T cells transiently co-transfected with LTα and GpL-sLTβ or GpL-sLTβ only encoding expression plasmids, washed four times by centrifugation (1 min, 13,000 rpm), resuspended in ice-cold PBS, and were finally resuspended in 50 µl of RPMI 1640 media supplemented with 0.5% FCS for the determination of GpL activity. For this purpose, cells were transferred to a black 96-well plate and 25 µl of the GpL assay solution (1.5 µM Coelenterazin (Carl Roth, Germany, 4094.3) in PBS) was added. Luciferase activity was immediately measured (1 s per well) using a PHOMO Photometer (Anthos Mikrosysteme, Germany).

#### Binding of GpL-tagged soluble receptor variants to membrane-bound TNF ligands

HEK293T cells transiently transfected with expression plasmids encoding LTα and memLTβ or empty vector (EV), or Flp-In-HEK293-mem(sc)LTα_2_β, Flp-In-HEK293-mem(sc)LTαβ_2_, and Flp-In-HEK293-EV or CHO and CHO-Δ(1-12)TNF cells were harvested and aliquoted in 1.5 ml safety-lock tubes (0.5–1.5 × 10^6^ cells/tube). Indicated cells were pretreated with 20 µg/ml TNF for 30 min at 37°C. Cells were incubated for 1 h (37°C) with supernatants containing TNFR1ed-, TNFR2ed-, and LTβRed-GpL. After removal of unbound molecules, cells were washed four times by centrifugation (1 min, 13,000 rpm) and cell-bound GpL activity was determined as described above. Specific binding was obtained by subtraction of the non-specific binding values, derived from the empty vector control cells, from the corresponding total binding values derived from the ligand-expressing cells. Data analysis was performed with the GraphPad Prism5 software.

### Flow cytometry

To control the success of transient and endogenous expression of TNF receptors and membrane-bound TNF ligands, cells were harvested and washed with PBS. Cells (0.3-2 × 10^6^ cells) were resuspended in PBS and were incubated for 1 h on ice with the PE-labeled antibody of interest or an appropriate PE-labeled isotype control antibody with the dilution/concentration recommended by the supplier. After removal of unbound antibodies by washing with PBS, cells were analyzed with an Attune NxT Flow Cytometer (Invitrogen, CA, USA).

### IL8 ELISA

Cells of interest were seeded in 96-well plates. Next day, the medium was replaced with fresh medium containing the reagents or stimulator cells of interest. After an additional day, supernatants were analyzed for IL8 expression using the BD OptEIA^TM^ human IL8-ELISA kit (BD Biosciences, NJ, USA).

### Determination of necroptosis and apoptosis

Fairly adherent responder cells were seeded in 96-well plates. The next day, responder cells (HeLa-RIPK3-FADD_KO_, Kym-1) were challenged with the stimulator cells of interest (e.g. mem(sc)LTα_2_β and mem(sc)LTαβ_2_ transfectants) overnight at a ratio of 1:1. Where indicated, cells were pretreated with 2.5 µg/ml CHX to sensitize for apoptosis. To prove that the reduced viability was due to necroptotic or apoptotic cell death, cells were rescued by adding 90 µM nec-1 to inhibit necroptosis or by adding 20 µM zVAD to prevent apoptosis. Induction of necroptotic and apoptotic cell death was furthermore substantiated by quantification of viable cells using crystal violet staining and by analyzing total cell lysates by western blot for S166 phosphorylation of RIPK1 and cleavage of caspase-8 and caspase-9, as well as PARP and β-Aktin. Before the final analysis, mem(sc)LTα_2_β and mem(sc)LTαβ_2_ transfectants, which poorly adhere to the cell culture plastic or the adherent responder cells, were removed by one wash with PBS.

## Supplementary information

supplemental data figures

supplemental data table
