# Supplementary material for: Membrane lymphotoxin-α2β is a novel tumor necrosis factor (TNF) receptor 2 (TNFR2) agonist
Source: Cell Death Dis. 2021 Apr 6;12(4):360. doi: 10.1038/s41419-021-03633-8 (PMC8024344; doi:10.1038/s41419-021-03633-8)
Supplement: Supplementary file 1 — supplemental data figures [file 41419_2021_3633_MOESM1_ESM.pdf]

Supplemental data

Membrane lymphotoxin-$\alpha_2\beta$ is a novel tumor necrosis factor (TNF) receptor 2 (TNFR2) agonist

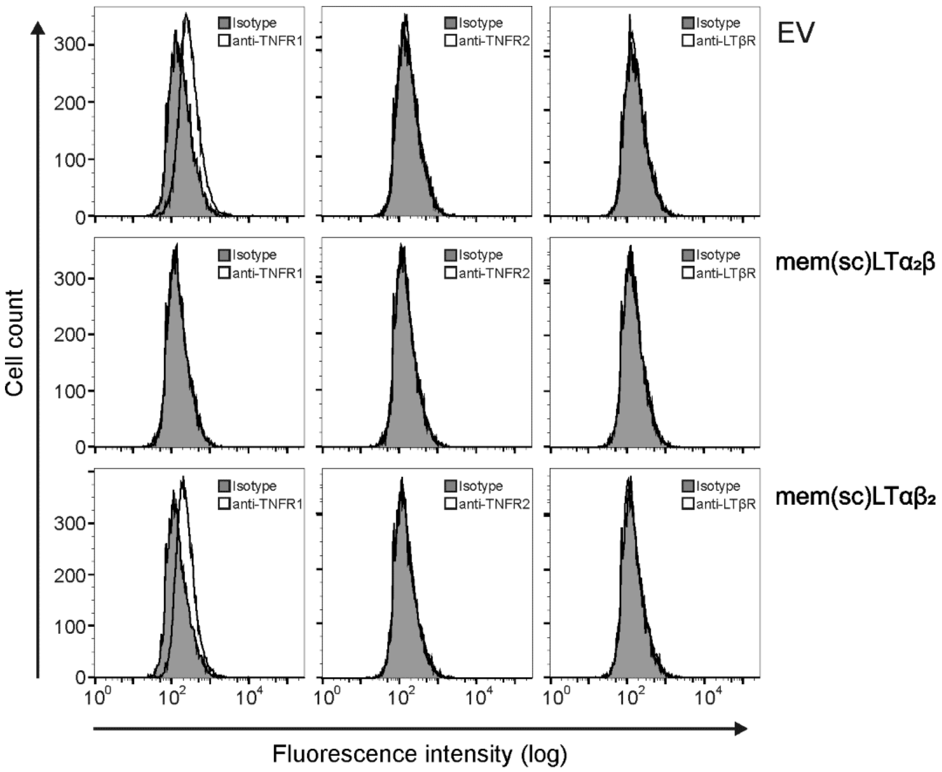

Supplemental Figure 1. Cell surface expression of TNFR1, TNFR2 and LTβR in Flp-In-HEK293 cells.

The indicated Flp-In-HEK293 transfectants were analyzed by flow cytometry with respect to the expression of TNFR1, TNFR2 and LTβR using PE-labeled anti-LTβR IgG1 (FAB629P, R&D, MN, USA), PE-labeled anti-TNF-R1IgG1 (FAB225P, R&D, MN, USA) and PE-labeled anti-TNF-R2 IgG2A (FAB226P, R&D, MN, USA) along with PE-labeled anti-mouse IgG1/IgG2A control antibodies (IC002P and IC002P, R&D, MN, USA). Please note, the lack of TNFR1 detection in the mem(sc)LT$\alpha_2\beta$ cells was regularly observed. The underlying reason is unclear. Possible explanations are counter selection against permanent TNFR1 activation due to mem(sc)LT$\alpha_2\beta$ triggered TNFR1 signaling or competition between mem(sc)LT$\alpha_2\beta$ and the anti-TNFR1 antibody for TNFR1 binding. In any case, it does not impact the use of the cells as mem(sc)LT$\alpha_2\beta$ expressing stimulator cells.

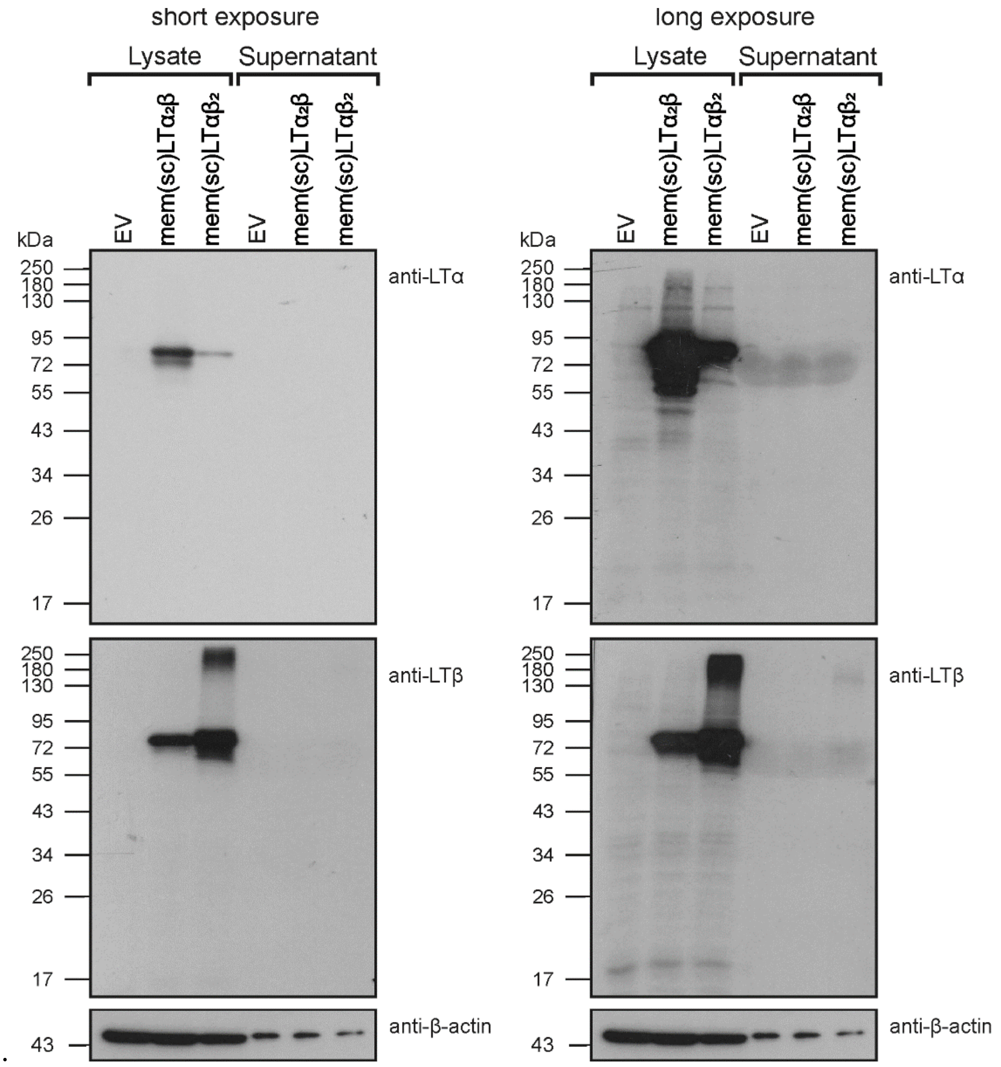

Supplemental Figure 2. Western Blot analysis of Flp-In-HEK293 cells stably expressing mem(sc)LTα$_2$β and mem(sc)LTαβ$_2$.

The indicated Flp-In-HEK293 transfectants were cultured for one day. Total cell lysates and supernatants were analyzed by western blotting using antibodies recognizing LTα, LTβ (anti-TNFβ (TNFβ = LTα) (E-6), sc-28345, anti-LTβ, MAB1684, R&D, MN, USA) and ß-actin. EV indicates empty vector transfected Flp-In-HEK293 cells.

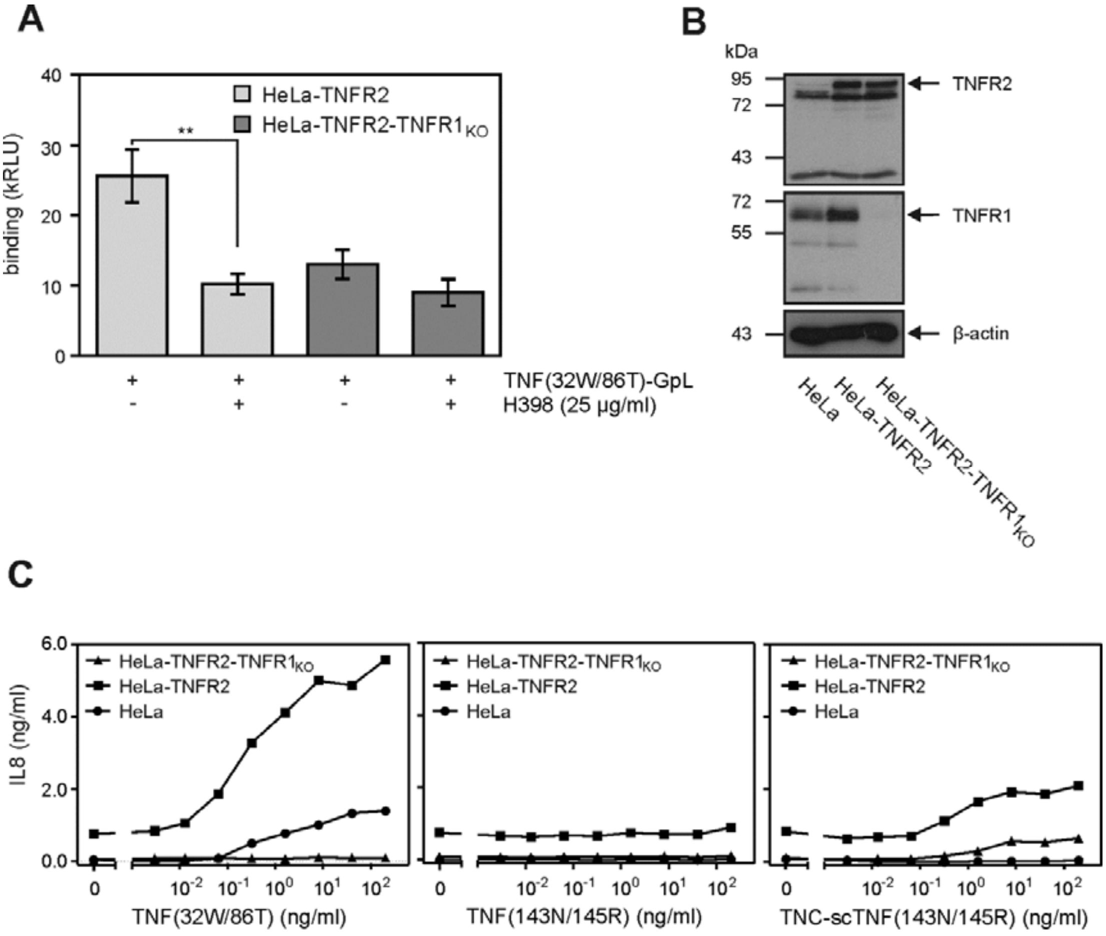

Supplemental Figure 3. Characterization of TNFR1-deficient HeLa-TNFR2 cells.

(A) Cells were incubated with 50 ng/ml of the TNFR1-specific TNF variant GpL TNF(32W/86T) in the presence and absence of the TNFR1 blocking antibody H398 (25 µg/ml). After one hour cell bound GpL activity was determined. **(B)** HeLa, Hela-TNFR2 and HeLa-TNFR2-TNFR1KO cells (1,5 x 10^6 cells each) were analyzed for TNFR1 and TNFR2 expression by western blotting using anti-TNFR1 and TNFR2 antibodies from Cell Signaling, MA, USA (3736S (TNFR1) and 3727S (anti-TNFR2). **(C)** The indicated cells were stimulated with the TNFR1-specific TNF variant TNF(32W/86T), the TNFR2-specific TNF variants TNF(143N/145R) and TNC-scTNF(143N/145R). Next day, cell supernatants were analyzed for IL8 production by ELISA. Please note, TNF(143N/145R) binds but do not activate TNFR2 while TNC-scTNF(143N/145R) binds and activates TNFR2 (18). All data shown are representative for at least 2 independent experiments. Please note, the conventional trimeric TNF mutant TNF(143N/145R) binds but does not activate TNFR2 while TNC-scTNF(143N/145R), which contains nine mutated TNF protomers, is a strong activator of TNFR2. (Rauert, H. et al. Membrane tumor necrosis factor (TNF) induces p100 processing via TNF receptor-2 (TNFR2). *J. Biol. Chem.* **285,** 7394-7404 (2010)).

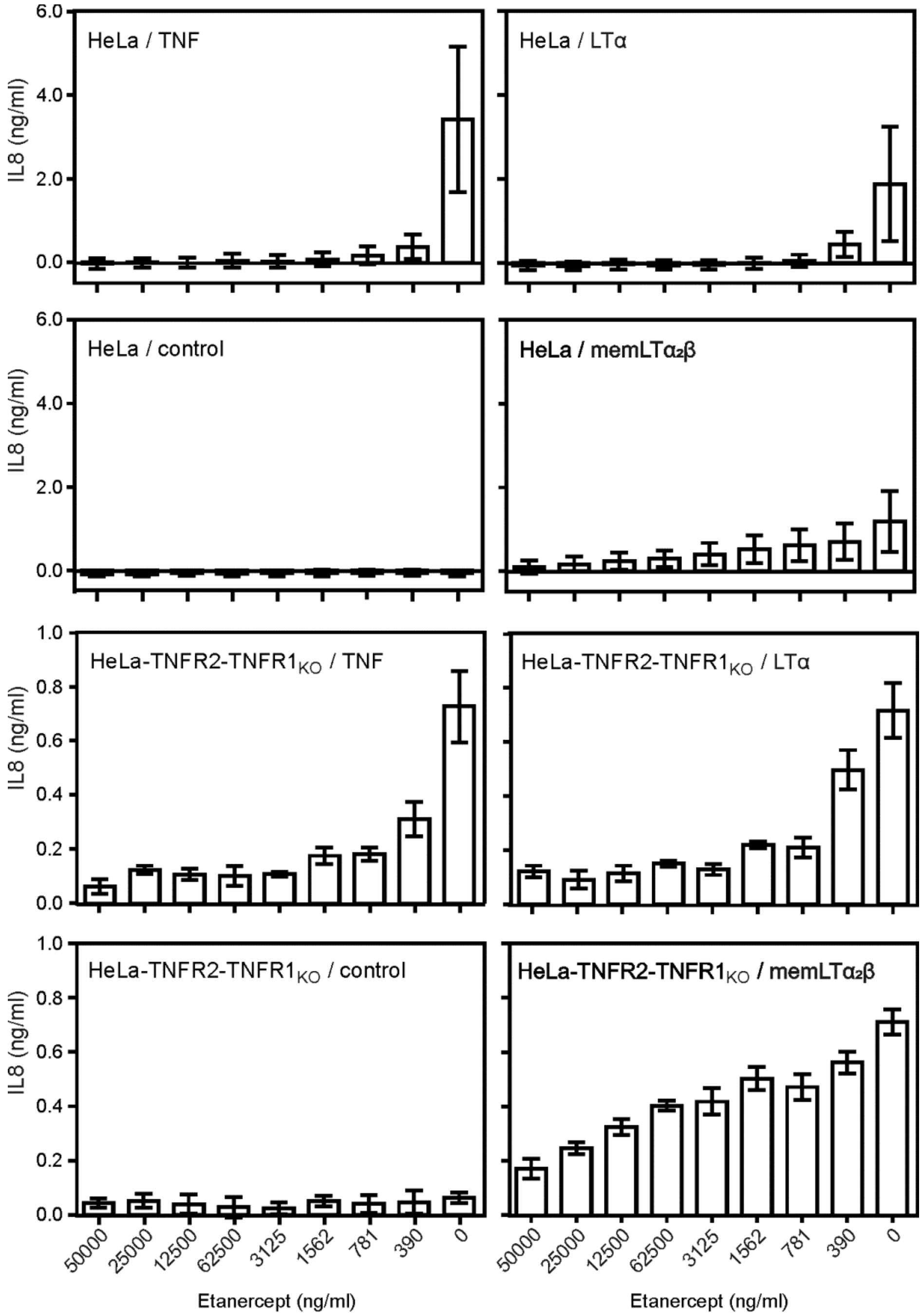

Supplemental Figure 4. Etanercept inhibits LTα, TNF and mem(sc)LTα$_2$β.

HeLa- and HeLa-TNFR2-TNFR1$_{KO}$ cells were challenged 1:1 (5 x 10^4 cells each) overnight with Flp-In HEK293 cells expressing mem(sc)LTα$_2$β or 50 ng/ml of LTα or TNF in the presence and absence of the indicated concentrations of Etanercept. IL8 production was determined by ELISA. One representative experiment of 3 independent experiments is shown.
