# Supplementary material for: Membrane lymphotoxin-α2β is a novel tumor necrosis factor (TNF) receptor 2 (TNFR2) agonist
Source: Cell Death Dis. 2021 Apr 6;12(4):360. doi: 10.1038/s41419-021-03633-8 (PMC8024344; doi:10.1038/s41419-021-03633-8)
Supplement: Supplementary file 2 — supplemental data table [file 41419_2021_3633_MOESM2_ESM.docx]

**Supplemental Table 1. Amino acid sequences of expression plasmids.**

| anti-TNFR1(13.7)-HC-Fab | L^a^ - QL - F^b^ - EF - V_H_ (US 2013/0251707 A1, Sequence 7) - GSRS - C_H_ (3U30_C^g^: aa 118-227) |
| --- | --- |
| anti-TNFR1(13.7)-LC | L^a^ - QL - F^b^- EF - V_L_ (US 2013/0251707 A1, Sequence 8) - GS - C_L_ (5EU7_C^g^: aa 105-213) |
| anti-TNFR2(C4)-IgG1-HC | ^18^ |
| anti-TNFR2(C4)-LC | ^18^ |
| anti-LTβR(BHA-10)-HC-Fab | ^17^ |
| anti-LTβR(BHA-10)-LC | ^17^ |
| TNFR1(ed)-GpL(w/o) | TNFR1 (NP_001056.1^h^: aa 1-211) - GSAGEF - F^b^ – GpL^f^ |
| TNFR2(ed)-GpL(w/o) | TNFR2 (NP_001057.1^h^: aa 1-257) - GSAGEF - F^b^ – GpL^f^ |
| LTβR(ed)-GpL(w/o) | LTβR (NM_002342.3^2^: aa 1-222) - GS - F^b^ - EF - F^b^ - LE – GpL^f^ |
| TNFR1(ed)-GPI | TNFR1 (NP_001056.1^h^: aa 1-211) - VD - GPI (NP_003832.3^h^: aa 157-259) |
| LTβR-GPI | LTβR (NP_002342.3^h^: aa 1-220) - VD - GPI (NP_003832.3^h^: aa 157-259) |
| TNF(32W/86T) | ^19^ |
| TNF(143N/145R) | ^19^ |
| TNC-scTNF(143N/145R) | ^19^ |
| memLTβ | LTβ (NM_002341^h^) |
| GpL-sLTβ | L^a^ - GS - GpL (AAG54095.1^h^) - GS - F^b^ - EF – LTβ^e^ |
| LTα | L^a^ - GS - F^b^ - EF - LTα^d^ |
| mem(sc)LTα_2_β | LTβ (NM_002341^h^) - LK^c^ – VEGGGS - LTα^d^ – GGGSVDYQF - LTα^d^ |
| mem(sc)LTαβ_2_ | LTβ (NM_002341^h^) - LK^c^ – VEGGGS - LTα^d^ - GGGVDYQF – LTβ^e^ |

L^a^ = Leader: MNFGFSLIFLVLVLKGVQCEVKLVPR

F^b^ = Flag: DYKDDDDK

LK^c^= Linker: GGGSGGGSGGGSGGGS

LTα^d^= LTα: aa 34-205 (NP_000586.2^2^)

LTβ^e^ =LTβ: aa 76-244 (NP_002332.1^2^)

GpL^f^ = GpL(w/o): aa 18-185 (AAG54095.1^2^)

^g^RCSB Protein Data Bank

^h^NCBI
